# Depressions with religious experiences

**DOI:** 10.1192/j.eurpsy.2022.567

**Published:** 2022-09-01

**Authors:** E. Gedevani, G. Kopeiko, O. Borisova, T. Vladimirova, E. Smirnova, V. Kaleda

**Affiliations:** 1 FSBSI Mental Health Research Center, Researching Group Of Specific Forms Of Mental Disorders, Moscow, Russian Federation; 2 Mental Health Research Centre, Department Of Youth Psychatry, Moscow, Russian Federation

**Keywords:** guilt and sinfulness, Depression, religious experiences, suicidal risks

## Abstract

**Introduction:**

Despite a significant number of studies devoted to the relationship between depression and religiosity, the diagnosis of depression in religious patients is complicated due to the insufficiently studied psychopathology and the peculiarities of the patient’s experiences.

**Objectives:**

To determine the specific features of psychopathology and phenomenology of depression, masked by a “religious facade”, for timely diagnostics and prevention of suicidal behavior.

**Methods:**

One hundred and fifteen religious (orthodox) inpatients (41 male, 74 female) with depression (F31.3, F31.4, F 32.1., F 32.2, F 33.1, F 33.2 according to ICD-10) were examined. Psychopathological method, HAM-D, SIDAS and statistical analysis were applied.

**Results:**

Five types of depression were specified, which differed in psychopathological structure and content of the religious experiences. Overvalued ideas of guilt and sinfulness were predominant in melancholic depressions, ideas of God-forsakenness and the loss of “living” faith - in apathetic. Depressions with overvalued doubts whether the right faith and confession has been chosen accompanied with anxiety, melancholy and apathy. It should be specially mentioned apathetic and melancholic depressions characterized by “spiritual hypochondria” with specific cenesto-hypochondrical symptomatology. Melancholic depressions characterized by high suicidal risk prevailed (65%) over the other depressions.

**Conclusions:**

Depressions masked by a “religious facade” often are not recognized due to specifical content, which results in lack of timely diagnostics and creates a high risk of suicidal behavior.

**Disclosure:**

No significant relationships.

